# Impact of Ptosis Surgery on Corneal Astigmatism in Eyes After Penetrating Keratoplasty: A Case Series

**DOI:** 10.7759/cureus.90667

**Published:** 2025-08-21

**Authors:** Masayuki Shimizu, Takashi Ono, Tetsuya Toyono, Takashi Miyai

**Affiliations:** 1 Ophthalmology, University of Tokyo, Tokyo, JPN

**Keywords:** corneal imaging, corneal tomography, corneal transplantation, ocular surface, ptosis

## Abstract

Background: Ptosis is a common complication of ophthalmological surgery and is known to alter the corneal shape. This study analyzed the effect of ptosis surgery on corneal astigmatism in patients with ptosis after penetrating keratoplasty using a Fourier harmonic analysis.

Methods: Patients who underwent ptosis surgery following penetrating keratoplasty at the University of Tokyo Hospital between 2017 and 2023 were included. A corneal tomographic analysis was performed using anterior segment optical coherence tomography before and three months after ptosis surgery.

Results: Seven eyes of seven patients (four men and three women; mean age = 72.9 ± 12.5 years) were included in the study. The Fourier analysis with a 6-mm diameter demonstrated that the anterior corneal spherical component, regular astigmatism, and asymmetry component did not change. Higher-order irregularity significantly decreased from 0.68 ± 0.27 D to 0.53 ± 0.25 D with surgery (p = 0.038). However, each component in the Fourier analysis for the posterior cornea remained unchanged.

Conclusion: Ptosis surgery altered the shape of the anterior corneal surface and reduced the incidence of corneal irregularities following penetrating keratoplasty.

## Introduction

Blepharoptosis, commonly known as ptosis, is characterized by drooping of the upper eyelids. It can be broadly classified as either congenital or acquired [1]. Among the acquired forms, ptosis is further categorized into aponeurotic, myogenic, neurogenic, mechanical, and traumatic types [2]. Environmental risk factors contributing to acquired ptosis include aging, prolonged use of contact lenses, and prior ocular surgeries [2]. The incidence of ptosis following ocular surgery varies widely, between 0% and 44%, and depends on the surgical and anesthetic techniques employed [3]. This variability underscores the complexity of ptosis as a postoperative complication, particularly after corneal transplantation procedures that can require multiple interventions, which increase the likelihood of eyelid droop.

Ptosis is not merely a cosmetic concern; it can significantly impair visual function by obstructing the visual axis and altering corneal curvature [4]. This obstruction can lead to decreased visual acuity and quality, necessitating surgical correction. Treatments for ptosis include levator advancement and internal levator/tarsus/Müller muscle resection approaches for aponeurotic ptosis. In cases of post-traumatic ptosis, where the levator palpebrae superioris or Müller muscle may be weakened or paralyzed, a frontalis muscle suspension is performed. The primary goal of ptosis correction surgery is to restore the eyelid to its normal position, thereby enhancing both the aesthetic and functional outcomes. Levator resection can effectively improve ptosis in patients following corneal transplantation [5].

Eyelid ptosis often induces a characteristic flattening of the superior cornea, with a corresponding steepening centrally. Surgical correction of ptosis has been shown to partially reverse these changes, restoring a more symmetrical corneal curvature. Corneal refractive power changes after ptosis surgery [6]. Despite advancements, the specific impact of ptosis surgery on corneal morphology, especially in eyes that have undergone penetrating keratoplasty (PK), remains inadequately researched. The cornea is critical for the refractive power of the eye; thus, any alteration in its shape can have profound implications for visual acuity and quality. Although existing literature provides some insights into the effects of ptosis surgery [5,6], its influence on corneal refractive changes in eyes with pronounced strong corneal astigmatism after PK has not been well documented.

Given these knowledge gaps, this study focused on evaluating the topographic changes induced by ptosis surgery in patients with significant corneal astigmatism following PK. By employing Fourier-harmonic analysis [7], we aimed to provide a detailed investigation of how ptosis surgery influences corneal shape and irregularity. Understanding these changes is crucial to optimizing surgical outcomes and improving the quality of life for patients with both ptosis and corneal astigmatism.

## Materials and methods

Patients

This retrospective, observational study was approved by the Institutional Review Board of the Research Ethics Committee of the University of Tokyo School of Medicine (Examination No. 2217) and adhered to the tenets of the Declaration of Helsinki. Informed consent was obtained from all the participants using the opt-out method.

Patients who underwent ptosis surgery and levator advancement for acquired aponeurotic ptosis at the University of Tokyo Hospital between January 2017 and December 2023 were included. Patients with insufficient topographic information of the eyes before and after ptosis surgery were excluded.

Corneal tomography was performed using anterior segment optical coherence tomography (AS-OCT) (CASIA2, Tomey Corporation, Tokyo, Japan) one month before and three months after surgery. Poor AS-OCT images were removed based on the image quality system application established by CASIA2. One eye from each patient was included in the analyses. Medical charts were retrospectively reviewed to obtain patient backgrounds, best-corrected visual acuity (BCVA), central corneal thickness, corneal astigmatism, and corneal power evaluated using a Fourier harmonic analysis based on AS-OCT.

Examinations

Corneal irregularities were evaluated using AS-OCT data. A Fourier harmonic analysis of the corneal topographic data was conducted as previously reported [7]. The reconstructed axial refractive power data based on corneal tomography were decomposed using a series of trigonometric components. The dioptric powers on the mire ring i, Fi(σ), were transformed into the trigonometric components of the form with the Fourier series harmonic analysis program included in the equipment, as follows:

$\displaystyle{\displaylines{F_i (\sigma)=a_0+c_1 cos⁡(\sigma-&alpha;_1 )+c_2 cos⁡2(\sigma-&alpha;_2 )+ c_3 cos⁡3(\sigma-&alpha;_3 )+⋯+c_n cos⁡n(\sigma-&alpha;_n ) }}$

Where *a0* is the spherical component of the ring, *2 × c_1_* is the asymmetric component (tilt or decentration), *2 × c_2_* is the regular astigmatism, and the summation of *c_3_-c_n_* refers to higher-order irregularity components. These four parameters were examined in the cornea within a 6-mm diameter using a built-in program in AS-OCT, and the values were averaged. The four components of the anterior and posterior cornea and their total values were assessed. Multiple examinations were performed for each patient with adequate fixation. During corneal imaging, the examiners either manually lifted the drooping eyelid or instructed patients to open their eyes widely to prevent eyelid obstruction. We eliminated the influence of the adherent eyelid as much as possible and extracted data with high reproducibility among the data of multiple examinations.

Surgical technique

All surgeries were performed and supervised by TT, an expert in eyelid surgery. Following subcutaneous anesthesia with xylocaine and epinephrine, the eyelid skin was incised. After the tarsal plate was exposed, the levator tendon was identified and stitched to the tarsal plate with a 6-0 polypropylene suture. The skin was sutured with 6-0 polypropylene, and ofloxacin ophthalmic ointment was applied. Postoperatively, ofloxacin ophthalmic ointment was applied five times daily for one week. The skin sutures were removed one week postoperatively.

Statistical analyses

After the normality test, a paired t-test was used to compare visual acuity, central corneal thickness, keratometric values, and the four components of the Fourier harmonic analysis. Statistical analyses were performed using the GraphPad Prism 9.5.1 (GraphPad Software, San Diego, CA). Statistical significance was set at p < 0.05. All values are expressed as mean ± standard deviation.

## Results

In total, seven eyes of seven patients (four men and three women) were included in the study. Their mean age was 72.9 ± 12.5 (range: 52 to 85) years, and their postoperative periods after PK were 8.7 ± 7.3 years (Table 1). The PK primary diseases comprised keratoconus in 42.9% (three eyes), corneal leukoma in 28.6% (two eyes), and herpetic keratitis in 28.6% (two eyes).

**Table 1:** Patient backgrounds. All values are expressed as mean ± standard deviation.

N	7 eyes of 7 patients
Sex (men:women)	4:3
Age (years)	72.9 ± 12.5
Postoperative periods after penetrating keratoplasty (years)	8.7 ± 7.3

All surgeries were performed without any complications. Margin reflex distance-1 (MRD-1) significantly improved from 0.64 ± 0.48 mm before surgery to 4.00 ± 0.82 mm after surgery (p < 0.001). The best-corrected visual acuity did not change three months after ptosis surgery from 0.57 ± 0.74 to 0.45 ± 0.71 (p = 0.36, Table 2). Based on the AS-OCT images, the central corneal thickness was the same as that before the ptosis surgery (p = 0.19, Table 2). Furthermore, there were no changes in the keratometric values such as steep keratometry, flat keratometry, and cylindrical power (p = 0.11, 0.12, and 0.63, respectively, Table 2).

**Table 2:** Visual acuity, corneal thickness, and keratometry of post-keratoplasty eyes before and after ptosis surgery. All values are expressed as mean ± standard deviation. Statistical significance was set at p < 0.05.

	Before surgery	3 months after ptosis surgery	P-value
Best-corrected visual acuity (logMAR)	0.57 ± 0.74	0.45 ± 0.71	0.36
Central corneal thickness (μm)	507.00 ± 42.16	496.00 ± 41.70	0.19
Steep keratometry (D)	45.61 ± 5.90	46.65 ± 5.40	0.11
Flat keratometry (D)	41.32 ± 5.83	42.37 ± 4.43	0.12
Cylindrical power (D)	4.29 ± 2.86	4.29 ± 2.99	0.63

**Figure 1:**
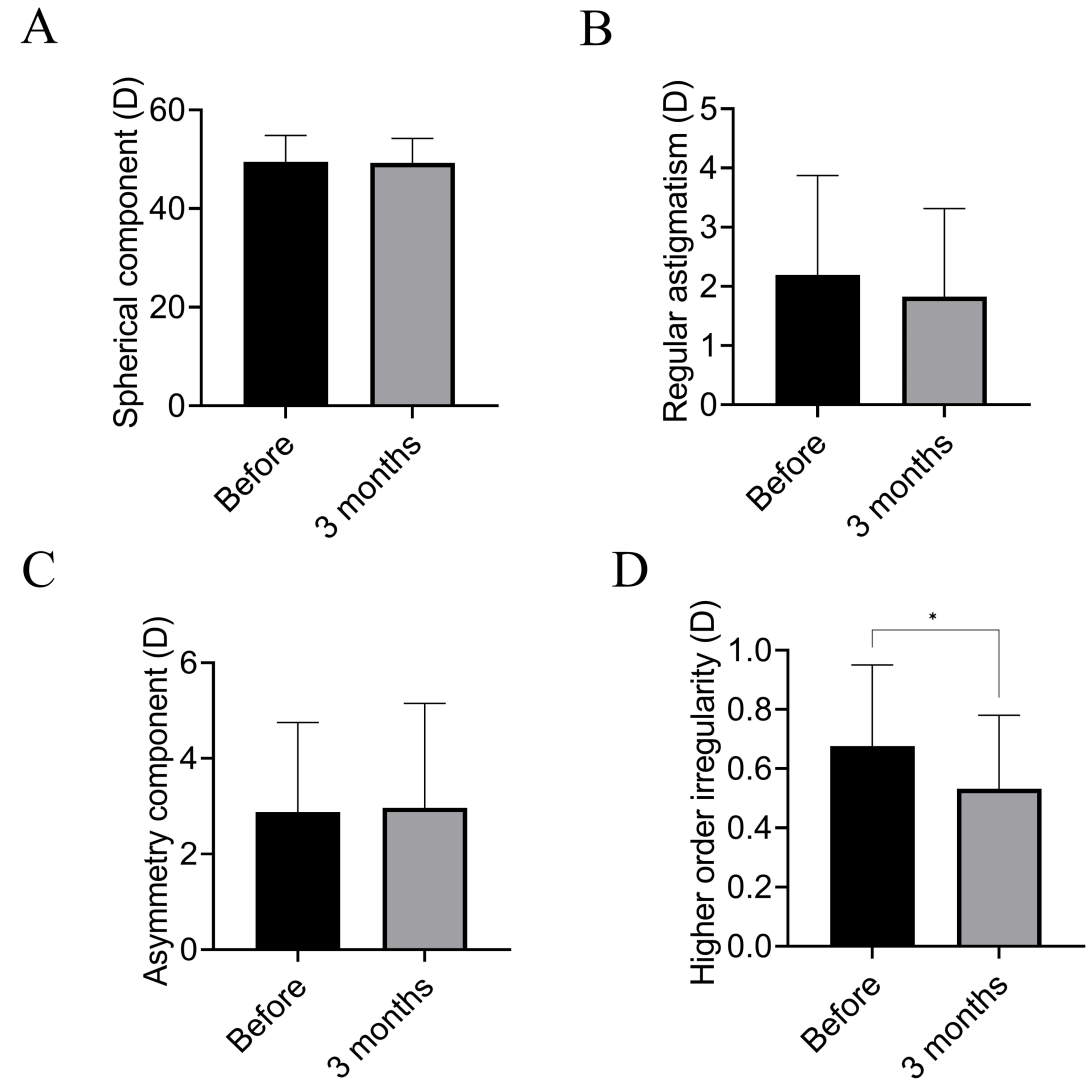
Fourier harmonic analysis of the anterior cornea before and after blepharoplasty. (A) Spherical component in Fourier harmonic analysis of the anterior cornea. No significant difference is observed between before and after surgery. (B) Regular astigmatism in Fourier harmonic analysis of the anterior cornea. No significant difference is observed between before and after surgery. (C) Asymmetry component in Fourier harmonic analysis of the anterior cornea. No significant difference is observed between before and after surgery. (D) Higher-order irregularity in Fourier harmonic analysis of the anterior cornea. Higher-order irregularity decreased after ptosis surgery (p = 0.038).

The Fourier harmonic analysis of the anterior cornea revealed that the spherical component, regular astigmatism, and asymmetric component did not change (Figures 1A-1C), although higher-order irregularity significantly decreased after ptosis surgery (p = 0.038) (Figure 1D).

**Figure 2:**
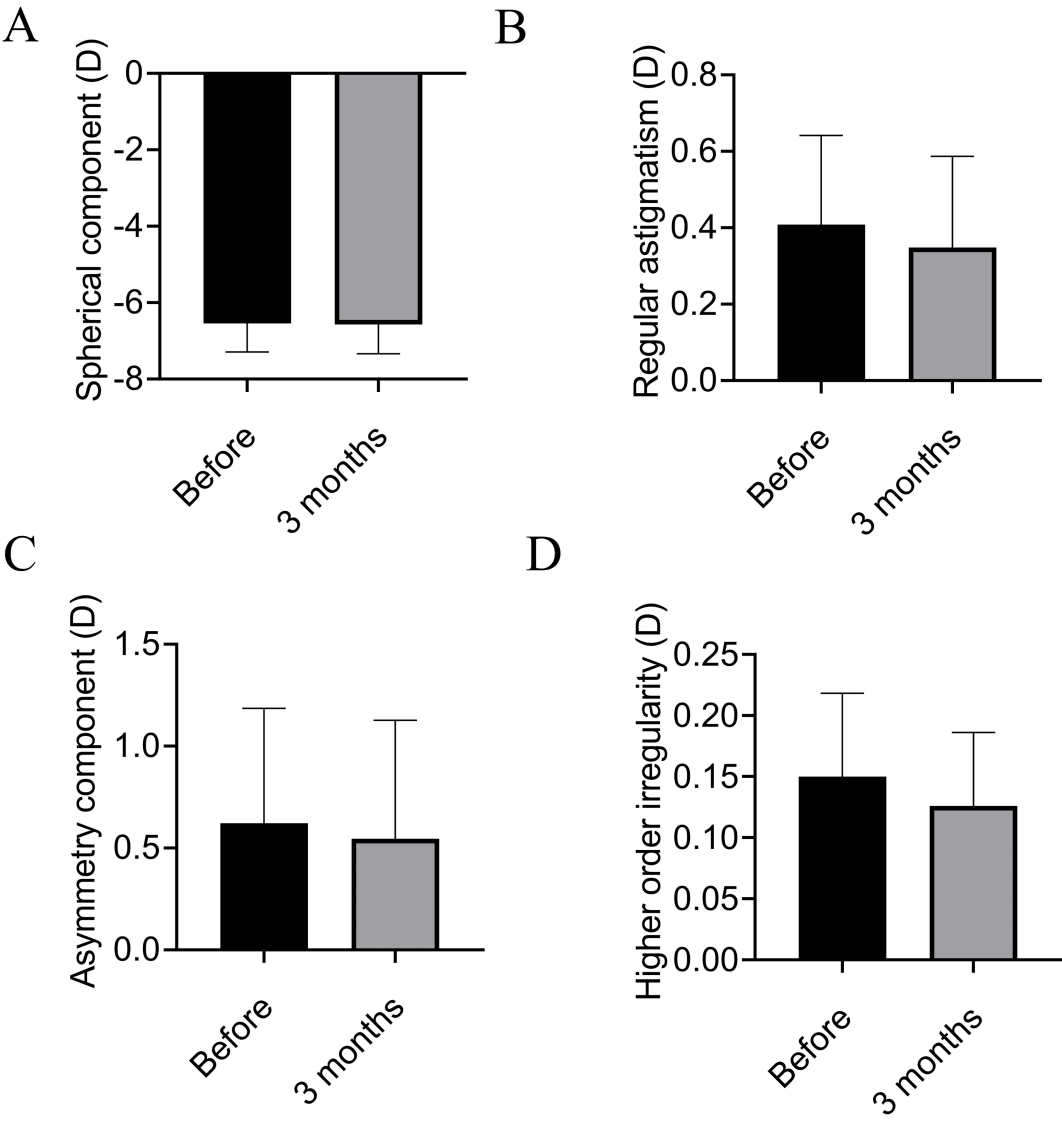
Fourier harmonic analysis of the posterior cornea before and after blepharoplasty. (A) Spherical component in Fourier harmonic analysis of the posterior cornea. No significant difference is observed between before and after surgery. (B) Regular astigmatism in Fourier harmonic analysis of the posterior cornea. No significant difference is observed between before and after surgery. (C) Asymmetry component in Fourier harmonic analysis of the posterior cornea. No significant difference is observed between before and after surgery. (D) Higher-order irregularity in Fourier harmonic analysis of the posterior cornea. No significant difference is observed between before and after surgery.

The analysis of the posterior cornea showed that the spherical component, regular astigmatism, asymmetric component, and higher-order irregularity did not change (Figure 2).

**Figure 3:**
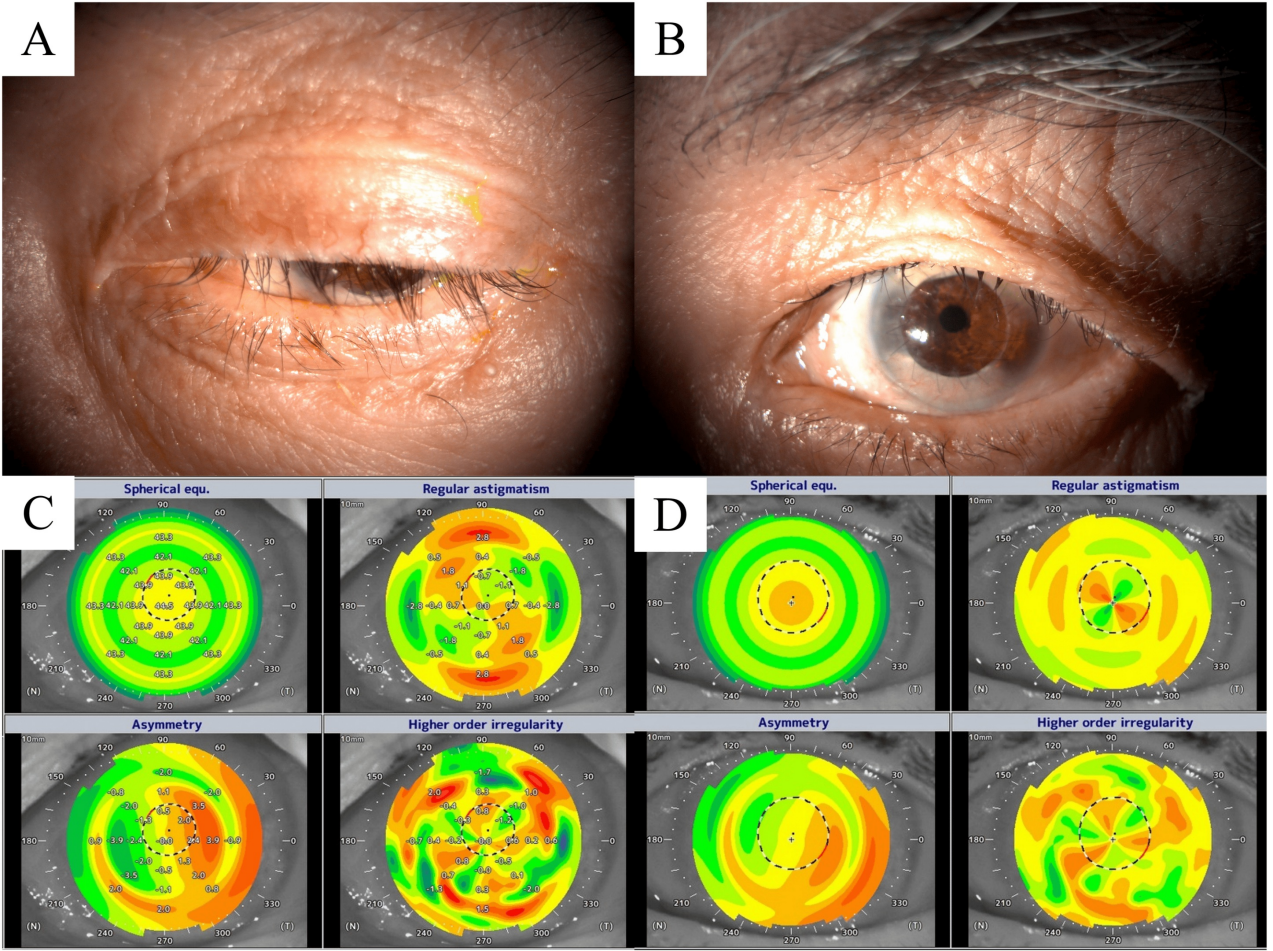
Representative case of blepharoplasty in eyes after penetrating keratoplasty. (A) A photograph of the anterior segment of the eye before ptosis surgery. The upper eyelid covers the cornea entirely. (B) A photograph of the anterior segment of the eye after ptosis surgery. The upper eyelid position is improved, and a clear cornea after penetrating keratoplasty is definably observed. (C) Results of the Fourier harmonic analysis in the eye before ptosis surgery. Higher-order irregularity illustrated right lower section is high and is represented in red color. (D) Results of the Fourier harmonic analysis in the eye after ptosis surgery. Strong higher-order irregularity represented in red color is decreased.

Representative images of the patients are displayed in Figure 3. An 80-year-old man underwent PK for corneal leukoma. Since this patient had previously undergone cataract surgery, blepharoptosis progressed after PK owing to aging and multiple ophthalmological surgeries (Figure 3A). After blepharoptosis surgery, ptosis improved, and a transparent corneal graft was clearly observed (Figure 3B). The lid no longer obstructed the visual axis. The Fourier harmonic analysis of the anterior cornea showed that the higher-order irregularities decreased postoperatively (Figures 3C, 3D).

## Discussion

This study evaluated the effects of ptosis surgery on corneal astigmatism in patients with ptosis after PK. The results demonstrated that ptosis surgery did not significantly alter the spherical component, regular astigmatism, or asymmetry, although it significantly reduced higher-order irregularities in the anterior cornea. This finding highlights the potential of ptosis surgery to refine corneal surface irregularities, which may contribute to improved visual quality in patients with significant corneal astigmatism after PK. In an eye with ptosis, the upper eyelid pressure flattens the upper cornea and steepens the central cornea [8,9], increasing asymmetric corneal astigmatism. Ptosis especially causes changes in the corneal shape. Ptosis surgery significantly reduces corneal astigmatism [6,10,11]. A reduction in irregularities could enhance the amount of light reaching the retina, thereby reducing visual distortions and potentially improving patient satisfaction [12]. Moreover, this suggests that the mechanical effects of eyelid surgery may extend beyond simple lid positioning and affect corneal biomechanics and surface regularity. To our knowledge, this is the first study focusing on corneal topographic changes induced by ptosis surgery in eyes with prior penetrating keratoplasty. Our results indicate that while standard astigmatism metrics remained unchanged, blepharoptosis surgery produced a measurable improvement in anterior corneal surface regularity.

The reduction in higher-order irregularities observed in this study suggests that ptosis surgery may enhance the optical quality of the cornea by smoothing out the anterior surface irregularities. This is particularly relevant for patients who have undergone PK because the corneal graft-host interface and subsequent healing can introduce irregularities that affect vision. Ptosis reportedly occurs in 6.7% of patients after anterior segment surgery [13,14]. PK is performed in patients with corneal opacity, trauma, infection, or poor prognosis with part transplantation [15-17]. Refraction changes significantly after PK, and the degree of postoperative astigmatism can significantly affect corrected visual acuity [18]. PK is performed to correct astigmatism in the cornea. By addressing these irregularities, ptosis surgery may offer complementary benefits beyond the primary functional and cosmetic objectives. This insight could inform preoperative discussions with patients and highlight the additional potential benefits of undergoing ptosis correction. Furthermore, understanding the biomechanical interplay between the eyelid and corneal surface can lead to more targeted surgical approaches that maximize both aesthetic and functional outcomes.

When eyelid pressure on the cornea is surgically removed, the corneal irregularity caused by ptosis is considered to improve [11,19]. Yamamoto et al. [6] reported that both anterior and posterior corneal astigmatism were significantly reduced after surgery for acquired ptosis. In the present study, only the higher-order irregular astigmatism component of the anterior surface was reduced, and the asymmetric component of the posterior surface also tended to decrease, suggesting that both the corneal surface and the entire structure were altered by the drooping eyelid. Our case with PK did not find any significant change in the regular astigmatism or keratometric values, and noted only a reduction in irregular astigmatism. This discrepancy is possibly caused by the fact that in post-PK eyes, the cornea’s regular astigmatism is largely determined by the graft and surgical sutures/scarring, making it less pliable to external eyelid pressure changes, whereas the more subtle irregular distortions are caused by the ptotic eyelid. The lack of change in regular astigmatism in our series may indicate that the overall graft curvature remained stable after ptosis repair, likely because those are determined by the transplant’s intrinsic properties. The improvement in higher-order irregularity suggests that eyelid pressure predominantly contributed to minor, irregular distortions of the anterior corneal surface, which were relieved by lifting the lid. We plan to increase the number of cases and re-examine these results in the future.

In contrast, the posterior cornea, which is less accessible and less influenced by external factors such as eyelid dynamics, might inherently possess a more stable morphology post surgery than the anterior cornea. This finding aligns with those of previous studies, indicating that anterior corneal changes are more pronounced in response to surgical interventions involving the eyelids [6]. This raises intriguing questions regarding the differential impact of mechanical forces on the anterior and posterior corneal surfaces. Investigating why the posterior cornea remains unaffected could provide deeper insights concerning corneal biomechanics and the potential protective role of the internal corneal structures.

Eyes with PK have large, irregular corneal astigmatism due to circumferential suture of the cornea [18], and corneal astigmatism is stronger than the value after endothelial keratoplasty as evaluated with a Fourier harmonic analysis [20]. Ptosis surgery for eyes that do not undergo corneal transplantation does not change the irregular astigmatism, although in eyes after corneal transplantation, the large irregular astigmatism after PK can improve. When cataract surgery is necessary for eyes after corneal transplantation for young corneal diseases, such as keratoconus and corneal dystrophy, it is advisable to carefully consider the power calculation of the intraocular lens after ptosis treatment because corneal refractive power can change after ptosis surgery. Further, in patients with a history of corneal transplant and coexisting significant ptosis, surgeons might consider addressing the ptosis before performing cataract surgery or other refractive procedures, to ensure corneal power has stabilized post-ptosis correction.

Limitations

Despite these results, this study was limited by its small sample size and retrospective design. The variability in patient demographics, underlying conditions leading to PK, and time elapsed since PK could have influenced the observed outcomes. Given the small sample, the statistically significant reduction in irregular astigmatism should be confirmed in a larger cohort to ensure this was not a spurious finding. Future studies with larger cohorts and a prospective design are warranted to confirm these findings and explore the long-term effects of ptosis surgery on corneal morphology and visual outcomes. Additionally, incorporating advanced imaging techniques and biomechanical assessments, such as using Corvis-ST® (Oculus Inc., St. Louis, MO), could further elucidate the underlying mechanisms by which eyelid position influences corneal shape. Understanding these dynamics may pave the way for innovations in surgical techniques and postoperative care to optimize patient outcomes.

## Conclusions

In conclusion, ptosis surgery appeared to effectively reduce higher-order irregularities of the anterior cornea in patients with ptosis after PK. This reduction can lead to an improved optical quality and potentially enhance visual outcomes. Although the primary objective of ptosis surgery is to restore eyelid function and aesthetics, its secondary benefits on corneal topography should not be overlooked. Further research is required to validate these findings and investigate the mechanisms underlying these changes.
